# Mesenchymal stromal cells and their secretory products reduce the inflammatory crosstalk between islets and endothelial cells

**DOI:** 10.1007/s12020-024-03975-1

**Published:** 2024-07-31

**Authors:** Rebecca Dewhurst-Trigg, Jessica Hopkinson, Sarah Richardson, Peter Jones, Chloe Rackham

**Affiliations:** 1 Exeter Centre for Excellence in Diabetes, Department of Clinical and Biomedical Sciences, University of Exeter, Exeter, UK; 2 Diabetes & Obesity, School of Cardiovascular and Metabolic Medicine & Sciences, London, UK

**Keywords:** Mesenchymal stromal cells, Secretome, Annexin A1, Islets, Endothelial cells

## Abstract

**Purpose:**

Preculturing isolated islets with Mesenchymal Stromal Cells (MSCs) improves their functional survival in vitro and subsequent transplantation outcomes in vivo. The MSC secretory product Annexin A1 (ANXA1) is a key modulator of MSC-mediated improvements in islet function. The current study aims to determine the influence of MSCs and defined MSC secretory products, including ANXA1, on the inflammatory crosstalk between isolated islets and Endothelial Cells (ECs), using in vitro models of the clinically-preferred intraportal islet transplantation niche.

**Methods:**

Islets were cultured alone, with MSCs, or with MSC secretory products and exposed to pro-inflammatory cytokines. Islet gene expression of C-C Motif Chemokine Ligand 2 (CCL2), C-X-C Motif Chemokine Ligand (CXCL)-10 (CXCL10) and CXCL1 were assessed by RT-qPCR. EC activation was induced with 100 U/ml TNF for 24 h. Islet-EC co-cultures were used to determine the influence of MSCs, or MSC secretory products on the inflammatory crosstalk between isolated islets and ECs. VCAM-1 and ICAM-1 expression were assessed at the mRNA and protein level in ECs, using RT-qPCR and immunofluorescence.

**Results:**

MSCs reduce pro-inflammatory cytokine-induced islet CCL2, CXCL10, and CXCL1 gene expression, which is partially mimicked by ANXA1. MSCs and ANXA1 have a similar capacity to reduce TNF-induced EC activation. Isolated islets exacerbate TNF-induced EC activation. Preculturing islets with MSCs reduces islet-exacerbated EC activation. ANXA1 reduces islet-exacerbated EC activation, when present during the islet preculture and islet-EC co-culture period.

**Conclusion:**

MSC-derived secretory factors, including ANXA1, may be used in islet transplantation protocols to target donor islet and host EC inflammation at the intraportal niche.

## Highlights

- MSCs and MSC-derived secretory factors, including Annexin A1, reduce EC activation.
- MSCs and MSC-secretory factors reduce islet expression of pro-inflammatory chemokines.
- MSCs and MSC-secretory factors reduce inflammatory crosstalk between islets and ECs.

## Introduction

Allogeneic islet transplantation is currently used to treat selected individuals with type 1 diabetes (T1D). However, its widespread adoption is restricted by the limited availability of donor islets, loss of islets during the in vitro preculture period [1, 2], and further extensive loss immediately post-transplantation when islet survival is compromised by the hypoxic, inflammatory host environment [3, 4].

Mesenchymal Stromal Cells (MSCs) are adult progenitor cells [5, 6] which can be expanded to clinically efficacious numbers in vitro. The minimal criteria for defining in vitro expanded MSCs is that they are plastic adherent, capable of in vitro differentiation into adipocyte, chondrocyte, and osteoblast lineages, and express CD73, CD90, and CD105, whilst lacking the expression of hematopoietic and endothelial markers [7, 8]. We and others have used both islet/MSC co-culture and co-transplantation strategies to demonstrate the capacity of MSCs to improve islet functional survival in vitro [9–13], and experimental transplantation outcomes in vivo [11, 12, 14–20]. However, the inherent heterogeneity of MSC populations presents problems in standardisation. Defining the mechanisms and secretory factors through which MSCs exert their therapeutic effects, in both pre- and post-transplantation protocols, will maximise their translational potential and facilitate significant advances within the field of beta cell replacement therapies.

MSCs secrete an array of immunomodulatory molecules (e.g., PGE2, TGF-β1, TSG6, ANXA1, and IDO-1 [15, 18, 21–23]) to influence host immune cells. We have previously used a quantitative mRNA screening approach to define a “cocktail” of MSC secretory products that are ligands for islet G-Protein Coupled Receptors (GPCRs) and target donor islets to improve their functional survival [21]. This screening approach demonstrated high expression levels of annexin A1 (ANXA1); stromal cell-derived factor-1 (SDF-1)/chemokine(C-X-C motif) ligand (CXCL) 12; and complement component C3a in both mouse and human MSC populations [9, 21, 24].

A defined MSC cocktail of ANXA1, SDF-1, and complement component C3a (C3a), improve the functional survival of donor islets following their ex vivo preconditioning, and subsequent experimental islet transplantation outcomes. Preculturing islets with this defined MSC cocktail reduces blood glucose in transplanted streptozotocin-diabetic mice, albeit not to the same extent as preculturing islets with MSCs [24].

It is likely that additive effects will be accomplished through using a dual-pronged MSC secretome-based approach to 1) preculture islets with a defined cocktail of MSC-secretory factors prior to transplantation [24]; and 2) deliver defined MSC-secretory factor(s) locally within the intraportal transplant niche to target islet cells and the host niche during the post-transplantation period.

One important event associated with the development of inflammation following clinical islet transplantation to the intraportal site is the disruption of the endothelial cell (EC) barrier in the vasculature. This leads to a cascade of additional events which activate cells of the innate immune system to amplify the subsequent cell-mediated immune response [25]. Islets transplanted via the clinically-preferred intraportal route lodge in the hepatic microcirculation where their local niche is the capillary endothelium [25]. Protocols which reduce the inflammatory status of donor islets and recipient ECs at the implantation site have significant potential to maintain the functional survival of transplanted islets, leading to improved clinical islet transplantation efficiency. We have begun to understand some of the crosstalk between MSCs and ECs [26]. However, very little is known about the secretory factors through which MSCs exert their anti-inflammatory properties on activated endothelium. In this study, we have used in vitro EC activation assays, as well as MSC-EC and islet-EC co-culture assays to investigate the inflammatory crosstalk between MSCs, islets, and ECs. We have shown that MSCs and the MSC secretory product, ANXA1 [9, 21], reduce the inflammatory status of both donor islets and host ECs. These studies suggest that MSC-derived secretory factors, including ANXA1, may be useful in islet transplantation protocols to target donor islet and host EC inflammation at the intraportal niche. Reducing the inflammatory crosstalk between islets and ECs at the intraportal implantation niche represents a crucial and novel MSC-mediated mechanism of immunomodulation to protect transplanted islets from the pro-inflammatory cytokine storm that prevails during the immediate post-transplantation period.

## Materials and methods

### Endothelial cell culture

The mouse microvascular EC line was generated by the immortalisation of mouse lung ECs (MLECs) with the polyoma virus middle T-antigen [27]. The cells were kindly provided by Prof. Stephen Robinson (University of East Anglia, Norwich, England). Cultures were seeded onto 1% gelatin-coated flasks and maintained in medium consisting of DMEM: Ham’s F12 supplemented with 0.1 mg/ml heparin (Merck, Hertfordshire, UK), 100 U/ml penicillin, 100 U/ml streptomycin, 4 mM glutamine (Gibco, Gaithersburg, Maryland) and 10% (vol./vol.) foetal bovine serum ((FBS) Merck), hereafter termed MLEC medium. Human Umbilical Vein Endothelial Cells (HUVECs) (Promocell, Heidelberg, Germany) were seeded into Nunclon 35 mm wells (Sarstedt Ltd, Leicester, UK) and cultured in EC medium ((Ready-to-use) (Promocell)), and supplemented with 100 U/ml penicillin, 100 U/ml streptomycin. All cells were maintained at 37 °C, 5% CO [2] and medium was replaced every 2–3 days.

### Endothelial cell activation assay

MLECs or HUVECs were seeded onto 13 mm glass coverslips (Scientific Laboratory Supplies (SLS), Nottingham, UK), which were precoated with 1% gelatin (Merck) for MLECs, at a density yielding monolayers of approx. 70% confluency within 24 h, or co-cultured with mouse or human MSCs, respectively, as below. After 24–48 h, MLECs or HUVECs were exposed to Tumor Necrosis Factor-αlpha (TNF) ((100 U/ml) (PeproTech, London, UK)), to induce the expression of adhesion molecules involved in leucocyte recruitment during the EC inflammatory response [26, 28, 29]. Mouse or human cytokines were used for MLECs and HUVECs, respectively. EC activation was assessed by quantifying the expression of the adhesion molecules Vascular Cell Adhesion Molecule-1 (VCAM-1) and Intercellular Adhesion Molecule-1 (ICAM-1), which are typically upregulated in the activated endothelium following exposure to inflammatory mediators [29]. RT-qPCR analysis of gene expression and/or immunofluorescence analysis of cell surface protein were used to assess EC activation [26, 29, 30].

### Co-culture of MSCs and ECs

MLECs or HUVECs were seeded into 35 mm wells at a density that yielded monolayers of approx. 70% confluency within 24 h culture in MLEC or HUVEC medium, respectively. In parallel, 100,000 mouse MSCs (Cyagen strain C57BL/6 bone marrow-derived MSCs; Generon, Slough, UK) or human MSCs (Stempro Human Adipose-derived; Life Technologies Ltd, Paisley, UK), were seeded on 6-well format transwell inserts with 0.4 µm pores (ThermoFisher Scientific, Paisley, UK) to reach a confluency of approximately 70% after 24 h. MSCs were cultured in DMEM supplemented with 1% (vol/vol) penicillin/streptomycin solution (Gibco) and 10% (vol/vol) FCS. After 24 h, the MSC transwell inserts were transferred to the 6-well plate containing MLEC or HUVEC monolayers (mouse or human MSCs, respectively), and MSCs and ECs were co-cultured. At this time point, 100 U/ml TNF was added to the MLEC or HUVEC monolayers, and the cells were co-cultured for another 24 h in MLEC or HUVEC medium. The cells were detached using acutase® solution (Merck) and collected separately for RNA extraction and subsequent cDNA synthesis, or paraformaldehyde-fixed for immunofluorescence staining.

### Islet isolation and culture

Mouse islets were isolated from male CD1 mice (Charles River, Margate, UK) by collagenase digestion (1 mg/ml; type XI; Sigma-Aldrich) followed by density gradient separation (Histopaque-1077; Sigma-Aldrich). After washing with RPMI-1640 medium, islets were picked into groups of 80-100 for culture alone, with MSCs, with recombinant ANXA1 alone (R & D Systems, Abingdon, UK), or with a defined cocktail of MSC-secretory factors (recombinant ANXA1 (5nmol/L), recombinant mouse SDF-1/CXCL12 (10 nmol/L), and recombinant mouse C3a (10nmol/L) [24]. For MSC co-culture, we utilised a direct-contact monolayer configuration to co-culture islets with MSCs, as previously described [11, 12]. Briefly, 100,000 MSCs of passage 7–12 were seeded into Nunclon™ 35 mm Petri dishes, forming a confluent monolayer of cells within 24 h. MSCs were cultured in DMEM supplemented with 1% (vol./vol) penicillin/streptomycin solution (Gibco) and 10% (vol./vol.) FCS. The medium was changed after 24 h, with removal of non-adherent cells. 80–100 freshly isolated islets were then added to each petri dish allowing direct cell-cell contact between the islets and pre-seeded MSCs. The medium was replaced with RPMI-1640 (supplemented with 10% (vol./vol.) FCS, 2 mmol/l glutamine, and 100 U/ml penicillin/0.1 mg/ml streptomycin). Islets cultured using the direct contact monolayer configuration formed loose attachments with the MSCs by 72 h but were removed for in vitro analysis by gentle pipetting. All animal procedures were approved by our institution’s Ethics Committee and carried out under license, in accordance with the UK Home Office Animals (Scientific Procedures) Act 1986.

### RT-qPCR analysis of gene expression

Total RNA was extracted from MSCs, ECs, and islets using RNeasy mini kits and RNase-free DNase sets (Qiagen, Manchester, U.K.), followed by reverse transcription into cDNAs using an Applied Biosystems high-capacity reverse transcription kit (Life Technologies Ltd, Paisley, UK). RT-qPCR of cDNAs was performed using QuantiTect primer assays and SYBR® Green (Qiagen), according to manufacturer’s instructions. Relative expression of mRNAs was determined against GAPDH as an internal reference and calculated by the 2-ΔΔCt method [21].

### Immunostaining and analysis of HUVEC monolayers

Intact HUVEC monolayers were immunostained with ICAM-1 and VCAM-1 antibodies for analysis of HUVEC activation. Briefly, HUVEC monolayers were washed in phosphate buffered saline (PBS) and fixed in 4% paraformaldehyde for 20 min before immunostaining at room temperature. HUVEC monolayers were blocked in 5% normal goat sera (NGS) in PBS for 30 min before incubation for 1 h with a rabbit anti-ICAM-1 antibody (1:100, Abcam, Cambridge, UK), washed in PBS and then incubated with an Alexa Fluor 488-conjugated goat anti-rabbit secondary antibody for 1 h (1:400, Life Technologies, Bleiswijk, Netherlands). HUVEC monolayers were subsequently incubated with a mouse anti-VCAM-1 antibody (1:50, Life Technologies), for 1 h, washed in PBS, and then incubated with an Alexa Fluor 555-conjugated goat anti-mouse secondary antibody for 1 h (1:400, Life Technologies). Cell nuclei were stained with 4’,6-diamidino-2-phenylindole (DAPI). Imaging of intact HUVEC monolayers was performed using a confocal microscope (Leica DMi8). Imaging was started at the top of the HUVEC monolayer and downwards through the EC monolayer. Confocal Z-stack projected HUVEC monolayer images, comprised of 11–18 × 0.68 µm slices, were produced to investigate HUVEC activation. Z-stack projected HUVEC monolayer images were analysed using HALO® v3.3, Cytonuclear FL V2.0.12 module (Indica Labs) to determine the percentage and mean fluorescence intensity (MFI) of ICAM-1 and VCAM-1 positive cells. MFI was quantified in HALO® as mean positive cytoplasm intensity.

### Statistical analysis

Statistical analysis used Student’s *t-*test or analysis of variance (ANOVA), as appropriate. A *p* value of *p* < 0.05 was considered significant. All data are expressed as means ± SEM. All statistical analysis were performed using GraphPad Prism version 10.

## Results

### MSCs reduce TNF-induced EC activation

**Fig. 1:**
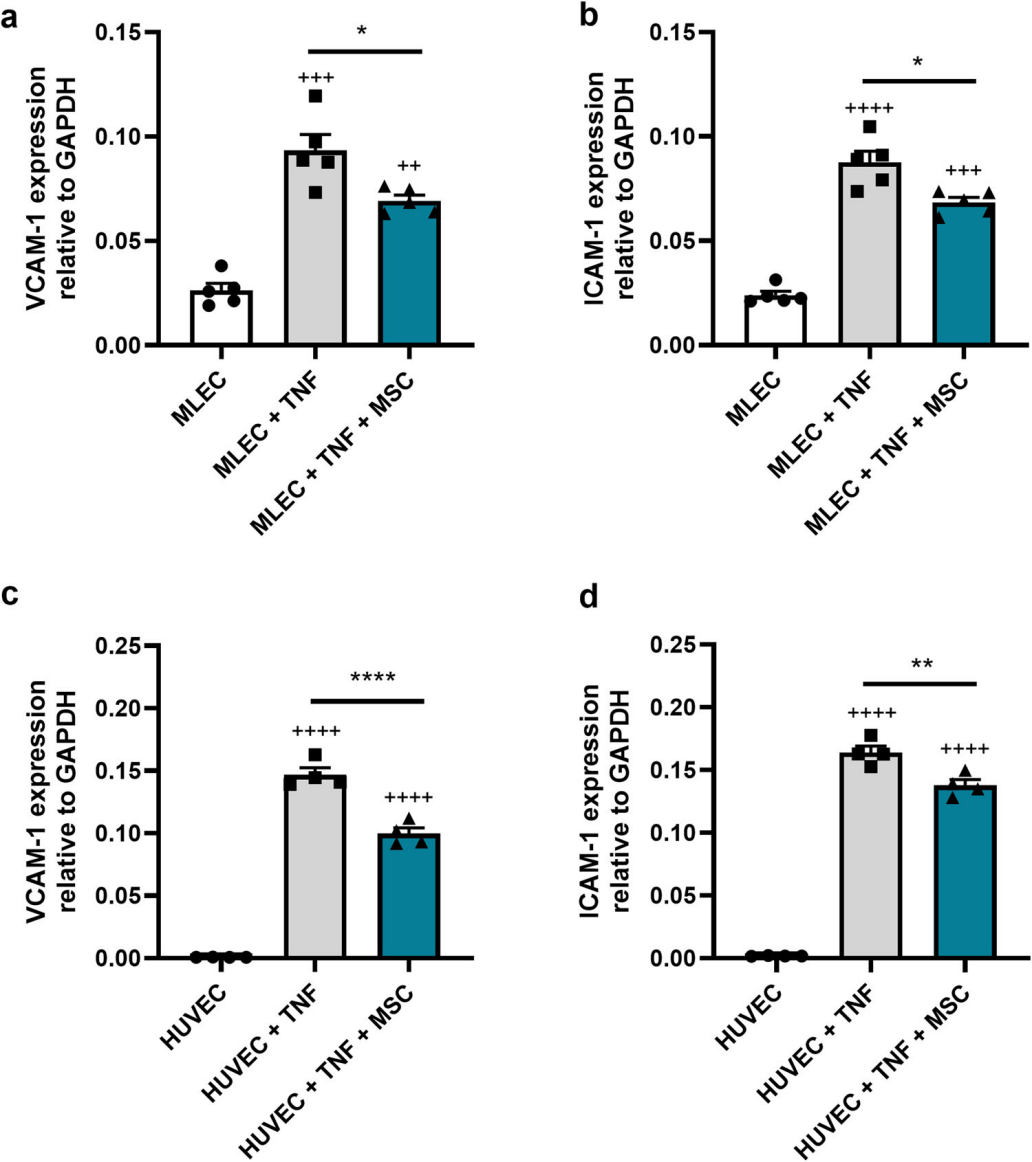
Mouse and Human MSCs reduce TNF-induced EC activation. Mouse MSCs reduce TNF-induced MLEC activation. MLEC monolayers were cultured without TNF (white bar) or exposed to 100 U/ml TNF and cultured alone (grey bars) or with mouse MSCs (teal bars) using an indirect contact transwell system (**a**, **b**), for 24 h. After 24 h adherent monolayer MLECs were harvested. VCAM-1 (**a**) and ICAM-1 (**b**) expression were quantified in MLEC lysates by RT-qPCR. *n* = 5 wells of 100,000 MLECs per condition, ++++*p* < 0.0001, +++*p* < 0.001, ++*p* < 0.01 vs. MLECs cultured alone in the absence of TNF, *p < 0.05 vs. MLEC exposed to TNF without mouse MSCs. Human MSCs reduce TNF-induced HUVEC activation. HUVEC monolayers were cultured without TNF or exposed to 100 U/ml TNF and cultured alone (grey bars) or with human MSCs (teal bars) using an indirect contact transwell system (**c**, **d**), for 24 h. After 24 h adherent monolayer HUVECs were harvested. VCAM-1 (**c**) and ICAM-1 (**d**) expression were quantified in HUVEC lysates by RT-qPCR. *n* = 4 wells of 100,000 orHUVECs per condition, ++++*p* < 0.0001 vs. HUVECs cultured alone in the absence of TNF, ****p < 0.0001 **p < 0.01, vs. HUVEC exposed to TNF without human MSCs. The *p* values were calculated using one-way ANOVA with Tukey’s Post Hoc Test

To determine the effect of mouse MSCs on activated MLECs, we performed co-culture experiments in which MLEC monolayers were exposed to 100 U/ml TNF, to simulate an inflammatory environment, and co-cultured with MSCs seeded on opposite sides of transwell inserts, for 24 h. To assess VCAM-1 and ICAM-1 expression levels in MLECs, we used RT-qPCR analysis of gene expression in MLEC lysates. Mouse MSCs reduce the expression of established markers of EC activation including VCAM-1 (Fig. 1a) and ICAM-1 (Fig. 1b). We further demonstrated that human MSCs reduce the expression of VCAM-1 (Fig. 1c) and ICAM-1 (Fig. 1d) in primary HUVECs exposed to 100 U/ml TNF. Given that the MSCs and ECs were separated by a transwell membrane, these observations suggest that the reductions in EC activation are likely to be mediated by MSC-derived soluble secretory factors.

### MSC-derived secretory factors reduce TNF-induced MLEC activation

**Fig. 2:**
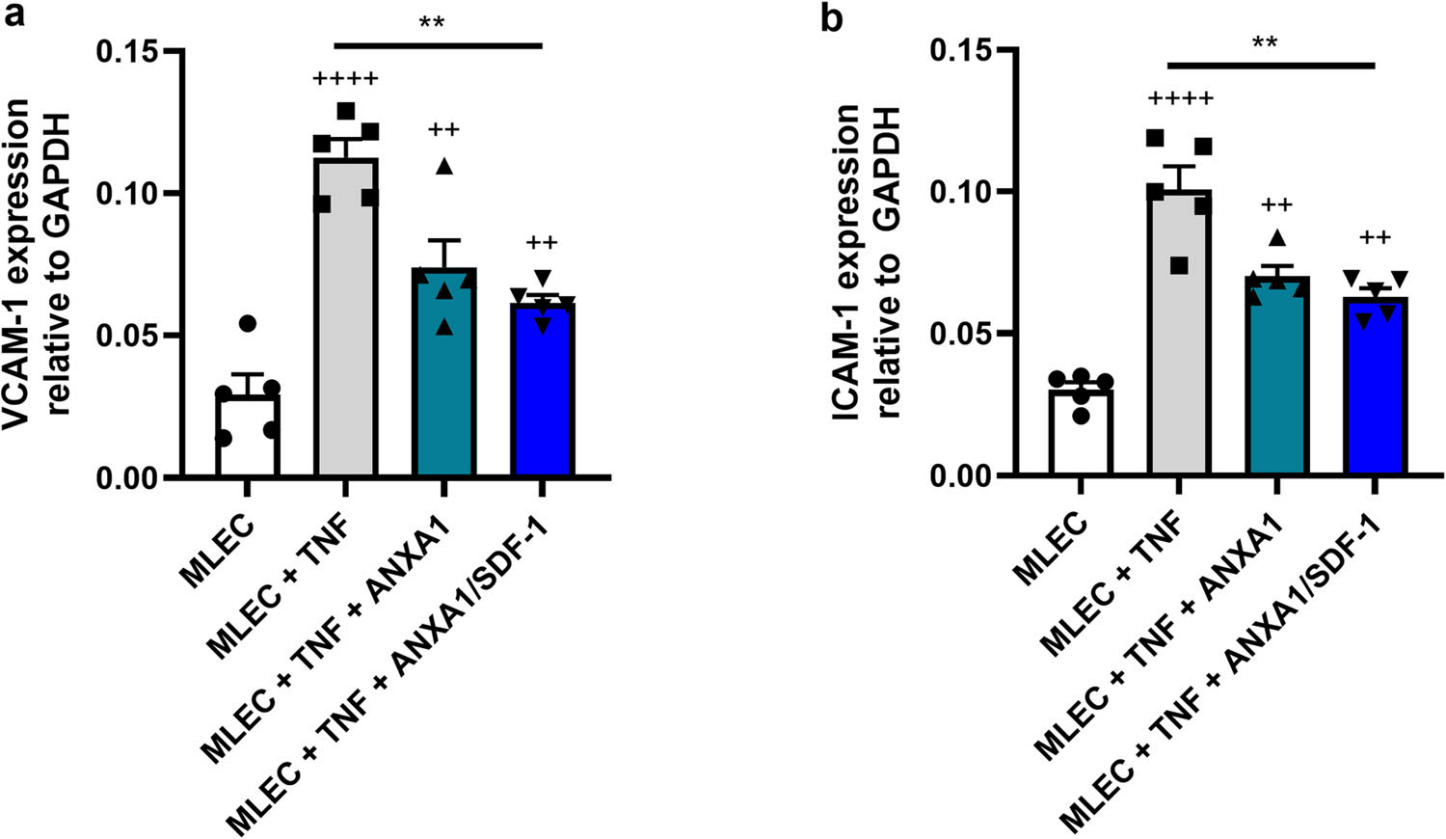
MSC-derived secretory factors reduce TNF-induced MLEC activation. MLEC monolayers were exposed to 100 U/ml TNF, and cultured either alone (grey bars), with 5 nM ANXA1 alone (teal bars), or with 5 nM ANXA1 + 10 nM SDF-1 (blue bars), for 24 h (**a**, **b**). After 24 h, adherent monolayer MLECs were harvested. VCAM-1 (**a**) and ICAM-1 (**b**) expression was quantified in MLEC lysates using RT-qPCR. Data are expressed as mean ± SEM, ++++*p* < 0.0001, +++*p* < 0.001, ++*p* < 0.01 vs. MLECs cultured alone in the absence of TNF (white bars), **p < 0.01 vs. MLECs exposed to TNF. The *p* values were calculated using one-way ANOVA with Tukey’s Post Hoc Test, *n* = 5

To determine whether MSC-derived secretory factors, ANXA1 alone or in dual combination with SDF-1, may be beneficial in post-transplantation strategies to reduce host EC activation, we exposed MLECs to TNF (100 U/ml) alone, or in the presence of ANXA1 or dual ANXA1/SDF-1, for 24 h. VCAM-1 and ICAM-1 expression levels in MLECs were assessed using RT-qPCR analysis of gene expression in MLEC lysates. ANXA1 treatment reduced TNF-induced MLEC expression of both VCAM-1 (Fig. 2a) and ICAM-1 (Fig. 2b). Treatment of MLECs with a dual combination of ANXA1/SDF-1 had no additive effect, with similar reductions in both VCAM-1 (Fig. 2a) and ICAM-1 (Fig. 2b) expression observed between MLECs treated with ANXA1 alone or ANXA1 together with SDF-1. Thus, recombinant ANXA1 alone treatment of MLECs is sufficient to mimic the effect of MSCs to reduce TNF-induced EC activation.

### ANXA1 reduces TNF-induced HUVEC activation

**Fig. 3:**
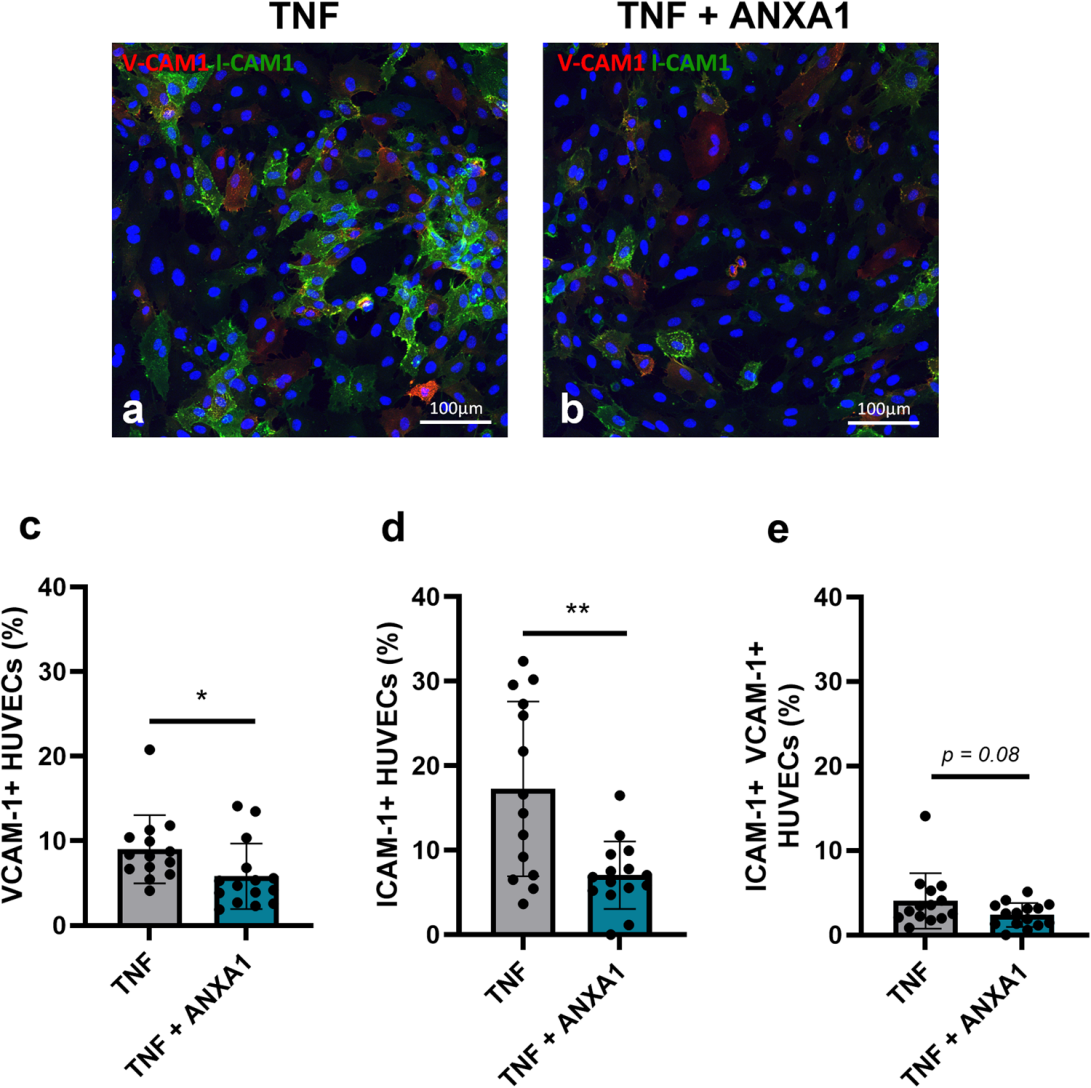
ANXA1 reduces TNF-induced primary HUVEC activation. Confocal micrographs showing representative images of Z-stack projected HUVEC monolayer images comprised of 7–15 × 0.68 µm slices, 20× magnification. HUVEC monolayers were exposed to 100 U/ml TNF-α alone (**a**), or to 100 U/ml TNF in the presence of 5 nM ANXA1 (**b**), for 24 h. Green and red indicate ICAM-1 and VCAM-1 immunostaining, respectively. The percentage of HUVECs immunopositive for VCAM-1 alone (**c**), ICAM-1 alone (**d**), or dual positive for both V-CAM1 and I-CAM1 (**e**) was assessed using HALO® v3.3, Cytonuclear FL V2.0.12 module (Indica Labs) image analysis software. 14-15 fields of view per condition assessed, *p < 0.05, ** *p* < 0.01 vs. HUVECs exposed to 100 U/ml TNF without ANXA1 treatment. The *p* values were calculated using Student’s t-test

We have shown that mouse and human MSCs reduce the expression of established markers of EC activation, including VCAM-1 and ICAM-1, using an immortalised MLEC line and primary HUVECs (mouse and human MSCs, respectively) and RT-qPCR analysis of gene expression (Fig. 1). We have further demonstrated that recombinant ANXA1 is able to mimic the anti-inflammatory effect of MSCs to reduce TNF-induced MLEC VCAM-1 and ICAM-1 gene expression (Fig. 2). In subsequent experiments, we have validated these findings using primary HUVEC monolayers exposed to 100 U/ml TNF (Fig. 3). ANXA1 treatment of TNF-exposed HUVECs reduced VCAM-1 and ICAM-1 gene expression (VCAM-1 expression relative to GAPDH: 0.13 ± 0.01 vs. 0.08 ± 0.01, TNF exposure alone vs. TNF exposure with ANXA1 treatment, *p* < 0.05, t-test; and ICAM-1 expression relative to GAPDH: 0.16 ± 0.02 vs. 0.12 ± 0.01, TNF exposure alone vs. TNF exposure with ANXA1 treatment, *p* < 0.05, t-test. We have further validated RT-qPCR measurements of gene expression using a semi-quantitative immunofluorescence approach to determine HUVEC expression of VCAM-1 and ICAM-1 at the protein level (Fig. 3).

Trypan blue assessment of cell viability showed that the percentage of viable HUVECs was comparable between control HUVECs cultured in the absence of TNF (95.46 ± 0.47%, *n* = 6), HUVECs exposed to 100 U/ml TNF for 24 h (97.56 ± 0.42%, *n* = 6), and HUVECs exposed to 100 U/ml TNF and treated with ANXA1 for 24 h (98.14 ± 0.92%, *n* = 6). Intact HUVEC monolayers were fixed and immunostained with VCAM-1 and ICAM-1 antibodies, without cell permeabilisation, to facilitate cell surface expression analysis. Confocal imaging of intact HUVEC monolayers cultured in the absence of TNF showed that HUVECs do not express cell adhesion molecules typically upregulated following exposure to pro-inflammatory stimuli. Thus, HUVECs cultured in the absence of TNF did not express VCAM-1 or ICAM-1, as expected (data not shown). Confocal imaging of TNF exposed HUVECs demonstrated cell-surface expression of VCAM-1 in approximately 10 percent of TNF exposed HUVECs and ICAM-1 in ~20% of TNF exposed HUVECs (Fig. 3a). Thus, 100 U/ml TNF enhances the expression of cell adhesion molecules associated with EC inflammation, without influencing cell viability. Semi-quantitative analysis of primary HUVEC monolayers confirmed that both VCAM-1 and ICAM-1 were significantly reduced at the protein level in ANXA1-treated HUVECs that had been exposed to 100 U/ml TNF (Fig. 3b). In keeping with this, the percentage of HUVECs positive for both VCAM-1 alone (Fig. 3c) and ICAM-1 alone (Fig. 3d) following a 24 h TNF exposure was reduced in ANXA1-treated HUVECs. There was also a strong trend (*p* = 0.08) towards a reduction in the percentage of double-positive ICAM-1/VCAM-1 cells in ANXA1-treated HUVECs (Fig. 3e). The MFI for both ICAM-1 and VCAM-1 was reduced in TNF exposed HUVECs treated with ANXA1 (ICAM-1 MFI: 71.33 ± 3.14 and 56.97 ± 4.67, TNF alone vs. TNF with ANXA1 treatment; VCAM-1 MFI: 65.75 ± 2.57 and 57.09 ± 1.19, TNF alone vs. TNF with ANXA1 treatment, *p* < 0.05, t-test, *n* = 14–15 fields of view). The total number of HUVECs per field of view was comparable between control TNF-exposed HUVECs and ANXA1 treated TNF-exposed HUVECs (TNF alone: 232.14 ± 15.63 cells; TNF + ANXA1: 214.27 ± 5.52 cells, *p* > 0.05, t-test).

### MSCs and their secretory factors reduce islet expression of pro-inflammatory chemokines

**Fig. 4:**
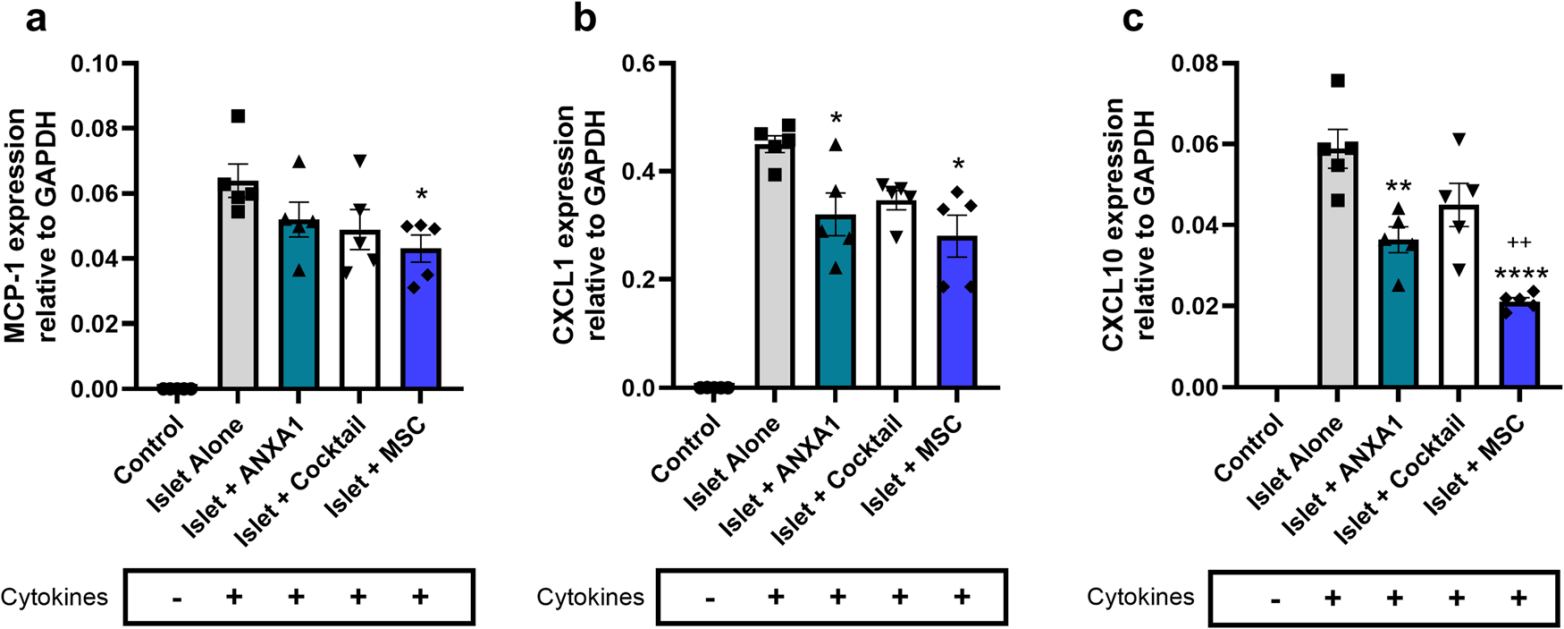
MSCs and their secretory factors reduce islet expression of pro-inflammatory chemokines. Control islets cultured without exposure to pro-inflammatory cytokines (control) express trace levels (Ct values > 30) of the pro-inflammatory chemokines CCL2/MCP-1 (**a**), CXCL1 (**b**), and CXCL10 (**c**), which are upregulated after 20 h exposure to pro-inflammatory cytokines (IL-1β (50 U/ml) and TNF (1000 U/ml)). Preculturing mouse islets with mouse MSCs (blue bars) reduces pro-inflammatory cytokine-induced expression of CCL2/MCP-1 (**a**), CXCL1 (**b**), and CXCL10 (**c**) compared to islets cultured alone (without MSC treatment) and exposed to pro-inflammatory cytokines (grey bar). Preculturing islets with ANXA1 (teal bars) partially mimics the capacity for MSCs to reduce pro-inflammatory cytokine-induced islet expression of pro-inflammatory chemokines, *n* = 5 groups of 100 islets per condition, *p < 0.05, **p < 0.01, ****p < 0.0001 vs. Islet Alone with cytokines, ++*p* < 0.01 vs. Islet + cocktail with cytokines. The *p* values were calculated using one-way ANOVA with Tukey’s Post Hoc Test

To determine whether MSCs and their secretory factors influence the inflammatory status of donor islets, isolated mouse islets were precultured alone, with MSCs, with recombinant ANXA1 alone or with a defined MSC-cocktail of recombinant factors (ANXA1/SDF-1/C3a [24]), for 48 hr and subsequently exposed to pro-inflammatory cytokines in low serum (2% FBS) RPMI culture medium for 20 h. Islet expression of pro-inflammatory chemokines was assessed by quantifying Monocyte Chemoattractant Protein-1 (MCP-1)/ C-C Motif Chemokine Ligand 2 (CCL2), C-X-C Motif Chemokine Ligand (CXCL)-10 (CXCL10) and CXCL1 gene expression using RT-qPCR. Control islets cultured without cytokines express trace levels (Ct values > 30) of pro-inflammatory chemokines CCL2/MCP-1 (Fig. 4a), CXCL1 (Fig. 4b) and CXCL10 (Fig. 4c). Preculturing mouse islets with MSCs reduces cytokine-induced islet expression of MCP-1 (Fig. 4a), CXCL1 (Fig. 4b) and CXCL10 (Fig. 4c). The MSC-mediated reduction in islet expression of pro-inflammatory chemokines is partially mimicked by ANXA1 and the MSC-cocktail of secretory factors [24], albeit not to the extent achieved with MSCs themselves.

### Preculturing islets with MSCs reduces islet exacerbated MLEC activation

**Fig. 5:**
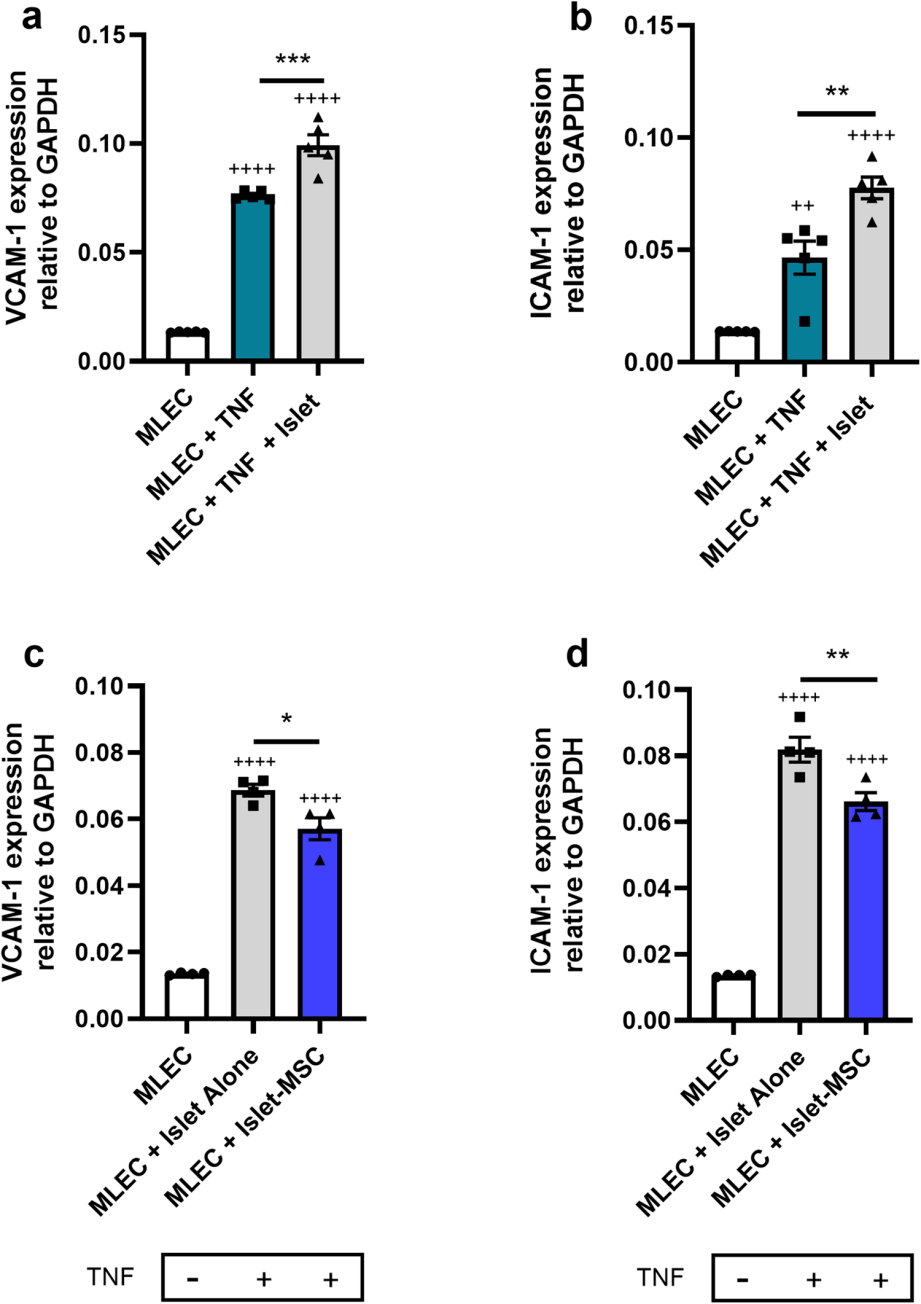
Preculturing islets with MSCs reduces islet exacerbated MLEC activation. **a**, **b** Isolated islets exacerbate TNF-induced MLEC activation. MLEC monolayers were exposed to TNF and co-cultured with islets. After 24 h islet-MLEC co-culture, islets and MLECs were separated, and adherent monolayer MLECs harvested. VCAM-1 (**a**) and ICAM-1 (**b**) expression was quantified in MLEC lysates by RT-qPCR, Data are expressed as mean ± SEM, ++++ *p* < 0.0001, ++ *p* < 0.01 vs. MLECs cultured alone in the absence of TNF, ***p < 0.001, ** *p* < 0.01. **c**, **d** MLEC monolayers were exposed to TNF and **c**o**-**cultured with islets that had previously been precultured alone (grey bars) or with MSCs (blue bars) for 48 h. After 24 h subsequent islet-MLEC co-culture, islets and MLECs were separated and harvested. VCAM-1 (**c**) and ICAM-1 (**d**) expression was quantified in MLEC lysates by RT-qPCR. ++++*p* < 0.0001 vs. MLECs cultured alone in the absence of TNF, *p < 0.05, **p < 0.01. Data are expressed as mean ± SEM, The *p* values were calculated using one-way ANOVA with Tukey’s Post Hoc Test, *n* = 4

We next sought to determine whether preculturing islets with MSCs influences MLEC expression of TNF induced VCAM-1 and ICAM-1. MLEC monolayers were exposed to 100 U/ml TNF, in the presence or absence of isolated islets, for 24 h. MLECs were co-cultured with islets using a direct contact configuration. Isolated mouse islets precultured without MSCs exacerbate TNF-induced MLEC expression of VCAM-1 (Fig. 5a) and ICAM-1 (Fig. 5b), after 24 h co-culture. Preculturing mouse islets with MSCs for 48 h prior to subsequent direct contact co-culture of islets and MLECs for 24 h with TNF exposure, reduced islet-exacerbated MLEC expression of both VCAM-1 (Fig. 5c) and ICAM-1 (Fig. 5d). The release of pro-inflammatory factors and chemokines by islets recruits immune cells and activates host ECs to amplify inflammation at the islet implantation site. We have shown that MSCs reduce islet expression of pro-inflammatory chemokines (Fig. 4) and that this correlates with MSC-mediated reductions in islet exacerbated MLEC expression of adhesion molecules involved in leucocyte recruitment during the EC inflammatory response (Fig. 5).

### Targeting host ECs with ANXA1 reduces islet-exacerbated MLEC activation

**Fig. 6:**
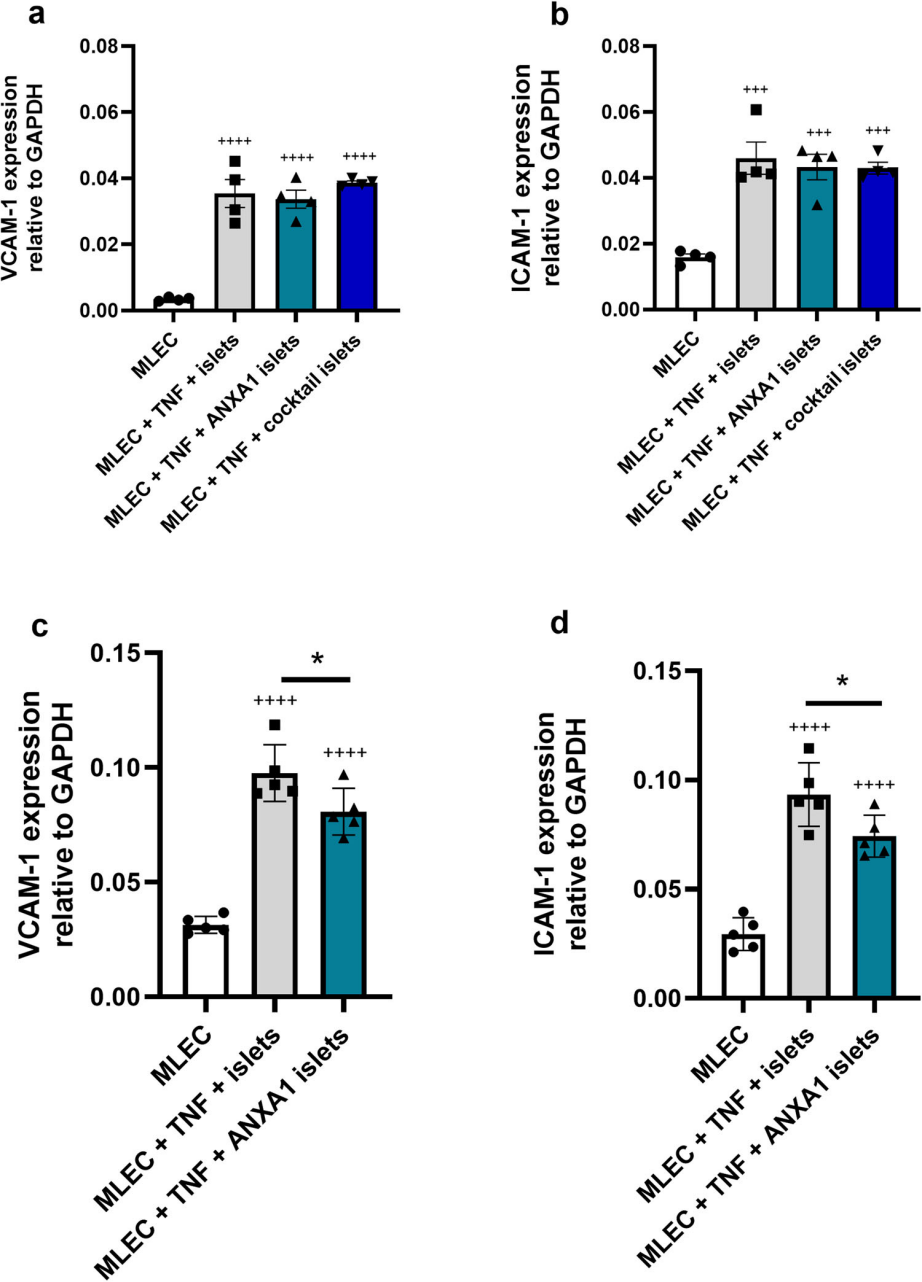
Targeting host ECs with ANXA1 reduces islet-exacerbated MLEC activation. **a**, **b**: Preculturing islets with ANXA1 or a cocktail of MSC secretory products, without subsequent ANXA1 treatment during TNF exposure of islet-MLEC co-cultures, does not influence islet-exacerbated MLEC activation. **a**, **b** MLEC monolayers were exposed to TNF and co-cultured with islets that had previously been precultured alone (grey bars) with ANXA1-alone (teal bars) or with an MSC-cocktail of ANXA1/SDF-1/C3a (blue bars) for 48 h. After 24 h subsequent islet-MLEC co-culture, islets and MLECs were separated and harvested. VCAM-1 (**a**) and ICAM-1 (**b**) expression was quantified in MLEC lysates by RT-qPCR. **a**, **b** +++*p* < 0.001, ++++*p* < 0.0001 vs. MLECs cultured alone in the absence of TNF. **c**, **d**: Preculturing islets with an MSC-cocktail of ANXA1/SDF-1/C3a, followed by treatment of islet-MLEC co-cultures with ANXA1 during TNF exposure of islet-MLEC co-cultures, reduces islet-exacerbated MLEC activation. MLEC monolayers were exposed to TNF and co-cultured with islets that had previously been precultured for 48 h alone (grey bars), or with an MSC-cocktail of ANXA1/SDF-1/C3a followed by ANXA1 treatment during the islet-MLEC co-culture period with TNF exposure (teal bars). Islets and MLECs were separated and harvested. VCAM-1 (**c**) and ICAM-1 (**d**) expression was quantified in MLEC lysates by RT-qPCR, ++++*p* < 0.0001 vs. MLECs cultured alone in the absence of TNF, *p < 0.05. Data are expressed as mean ± SEM, The *p* values were calculated using one-way ANOVA with Tukey’s Post Hoc Test, *n* = 5

Preculturing mouse islets with ANXA1 or a defined MSC-cocktail of secretory factors (ANXA1/SDF-1/C3a) [24] for 48 h, prior to a subsequent 24 h islet-MLEC co-culture and exposure to TNF, does not influence the induction of MLEC VCAM-1 (Fig. 6a) or ICAM-1 (Fig. 6b) expression. Thus, neither preculture of islets with ANXA1-alone or the MSC-cocktail mimic the beneficial effects of MSC preculture to reduce islet exacerbated MLEC activation. However, preculturing islets with the MSC-cocktail for 48 h, followed by ANXA1 treatment during the 24 h TNF exposure of islet-MLEC co-cultures does significantly reduce MLEC expression of VCAM-1 (Fig. 6c) and ICAM-1 (Fig. 6d).

## Discussion

The current study focused on understanding the functional effects of MSCs and their secretory factors on the inflammatory crosstalk between islets and the capillary endothelium. The hepatic portal vein is the preferred clinical islet transplantation site. MSC-based strategies that limit the inflammatory crosstalk between donor islets and host ECs have significant potential to reduce extensive islet cell death in the immediate post-transplantation period and prolong the functional survival of the islet graft.

Previous studies have shown that human MSCs reduce pro-inflammatory cytokine-induced HUVEC activation [26]. We have used an immortalized MLEC line to demonstrate similar mouse MSC-dependent reductions in TNF-induced MLEC activation. We have further adapted this in vitro model to incorporate isolated mouse islets, using a direct contact co-culture configuration, to mimic the intraportal transplantation niche. Our initial studies demonstrated that mouse MSCs reduce TNF-induced MLEC expression of VCAM-1 and ICAM-1; adhesion molecules typically upregulated in the activated endothelium following exposure to pro-inflammatory mediators [28, 29]. Identification of MSC-derived secretory factors that mimic the function of MSCs to reduce the inflammatory status of donor islets and host ECs is key to enabling the beneficial properties of MSCs to be incorporated into clinical islet transplantation protocols. This is because co-infused islets and MSCs disperse throughout the portal vasculature and are therefore unlikely to engraft together, with islets lodging in the hepatic microcirculation, whereas MSCs will most likely pass through the liver and engraft in the lung microcirculation [31]. Co-delivering effective numbers of MSCs with the islet graft is not practical [32]. An alternative strategy is to identify secretory factors, including anti-inflammatory, immunomodulatory peptides/proteins, that may be incorporated into biomaterials that enable nanocoating [33, 34] of the islets to ensure localised delivery of the specified secretory factors to the islet implantation site within the portal vasculature.

We previously reported that the MSC-derived secretory factor, ANXA1, directly influences islet beta cells to improve insulin-secretory function [9, 21]. The current study shows that ANXA1 also targets host ECs to reduce their activation and the inflammatory crosstalk between islets and ECs. ANXA1-dependent effects of reducing host EC inflammation were consistently observed in both MLECs and primary HUVECs, using the same therapeutic dose of 5 nM ANXA1, which targets mouse and human islets to improve their insulin-secretory function and confer protection from cytokine-induced islet apoptosis [9, 21].

We previously demonstrated that preculturing islets with a dual combination of MSC-secreted islet-GPCR ligands, ANXA1 and SDF-1, induces sustained improvements in islet beta cell insulin secretory function; an effect that is superior to that observed when preculturing with ANXA1 alone [24]. SDF-1 reduces thapsigargin or streptozotocin-induced beta cell apoptosis, emphasising a key pro-survival role of SDF-1 [35, 36]. In the current study, we have demonstrated no additive effect of SDF-1 on the ANXA1-dependent reductions in EC activation. Notably, no detrimental influence of SDF-1 on either donor islet or EC inflammation was observed. Whilst we have demonstrated no added benefit in terms of host EC inflammation, SDF-1 has been shown to influence host adaptive immune cell responses to transplanted islets. Thus, SDF-1 repels cytotoxic CD8+ T cells from infiltrating the islet graft whilst attracting protective regulatory T cells, and coating islets with SDF-1 delays allogeneic graft rejection [37]. Thus, incorporation of both ANXA1 and SDF-1 into biomaterials that enable localised delivery to the graft niche offers the potential to target both early EC inflammatory responses and longer term adaptive immune responses to transplanted islets.

Islets express a number of “danger signals” in response to inflammation, including MCP-1/CCL-2 [38], which is a key indicator of islet inflammation and is positively associated with other islet-expressed pro-inflammatory chemokines and cytokines (“isletokines”), such as CXCL1 and CXCL10 [39]. MCP-1 expression in donor islets is further associated with poor islet allograft outcomes in individuals with T1D [40, 41]. Pro-inflammatory chemokines recruit immune cells, resulting in further proinflammatory cytokine production from the infiltrating cells and the activation of transcription factors in beta cells to sustain and amplify inflammatory signalling pathways leading, ultimately, to the loss of beta cells [36]. The current study shows that preculturing islets with MSCs reduces the expression of key isletokines, typically upregulated following exposure to pro-inflammatory cytokines.

The release of pro-inflammatory factors by donor islets activates host ECs to amplify inflammation at the intraportal implantation site, which in turn contributes to extensive islet cell death [25]. We have shown that isolated islets exacerbate the inflammatory status of ECs as demonstrated by RT-qPCR analysis of VCAM-1 and ICAM-1 in EC monolayers harvested after direct contact co-culture with isolated islets. We have further demonstrated that the elevated expression of VCAM-1 and ICAM-1 is reduced when islets are co-cultured with MSCs, which is likely to be related to the inflammatory status of the islets themselves. Accordingly, we have shown that the MSC-dependent reduction in islet-expressed pro-inflammatory mediators is associated with a reduction in subsequent islet-exacerbated EC activation. The MSC-mediated reduction in islet expression of pro-inflammatory chemokines is partially mimicked by the MSC secretory product, ANXA1, albeit not to the extent achieved with MSCs themselves. Thus, preculturing islets with ANXA1 or the MSC-cocktail, did not mimic the effects of MSC preculture of islets to reduce subsequent islet exacerbated MLEC activation. In contrast, preculturing islets with the MSC-cocktail followed by ANXA1 treatment during the islet-EC co-culture with TNF significantly reduced both VCAM-1 and ICAM-1 expression in MLECs.

## Conclusion

In summary, the MSC secretory product, ANXA1, partially mimics the function of MSCs to reduce islet expression of pro-inflammatory chemokines and effectively recapitulates the effect of MSCs to reduce EC activation. The use of defined MSC-derived secretory factors to improve islet function and survival has obvious translational advantage, negating the many safety and regulatory concerns of incorporating MSCs into clinical transplantation protocols. Our studies suggest that additive effects to reduce the inflammatory crosstalk between donor islets and host ECs will be achieved through using a dual-pronged approach to; (1) incorporate ANXA1 into a defined MSC cocktail of secretory factors used to preculture islets for 24–48 h before transplantation [24]; and (2) co-delivery of ANXA1 with the islet graft to enable localised and more sustained targeting of both donor islets and host ECs during the early post-transplantation period. Reducing the inflammatory crosstalk between islets and ECs at the intraportal implantation niche represents a crucial and novel MSC-mediated mechanism of immunomodulation to protect transplanted islets from the pro-inflammatory cytokine storm which destroys as much as 70% of transplanted islets in the immediate post-transplantation period, thus limiting clinical efficacy.
